# TuberIndex 1.0, a dataset of ecological interactions from five centuries of French literature on Tuberaceae

**DOI:** 10.1038/s41597-026-07097-3

**Published:** 2026-03-26

**Authors:** Montan Gautier, Elisa Taschen, Nicolas Lescureux, Franck Richard

**Affiliations:** 1 UMR Centre d’Ecologie Fonctionnelle et Evolutive, Université Montpellier-CNRS-EPHE-IRD, Montpellier, Occitanie France; 2 Pépinières Tenoux, Valdoule, Provence-Alpes-Côte d’Azur France; 3 UMR Eco&Sols, CIRAD, INRAE, IRD, Institut AgroMontpellier, Université Montpellier, Montpellier, Occitanie France

**Keywords:** Ecological networks, Agroecology, Agriculture, History, Biodiversity

## Abstract

True truffles include the most prized mushrooms in the world and some of the most studied species model for mycorrhizal ecology. Despite the recurring difficulties encountered in isolating and cultivating truffle mycelium, a wealth of literature has been dedicated to the biology and ecology of these fungi since the early 19th. Here, we compile French-language literature on Tuberaceae, much of which remains underexplored despite its scientific potential. We report ecological interactions between truffles, plants, and other fungi. We present TuberIndex 1.0, an open-access dataset compiling five centuries of literature on Tuberaceae and their ecological interactions. The dataset indexes 493 documents and reports 3,508 records of interactions involving 26 truffle taxa, 418 plant taxa, and 53 fungal taxa. A central objective of the dataset is to facilitate future research by providing a valuable resource on truffle interactions, while also supporting innovative agricultural practices.

## Background & Summary

Truffles are among the most refined and prized delicacies enjoyed by gourmets worldwide^1^. Since the beginning of the 19th century, no other mushrooms have been the subject of such a continuous and sustained effort by scientists, foresters and naturalists to understand its biology and ecology^2^. As a result, truffles occupy a unique place in our scientific knowledge of fungal ecology and plant physiology. Notably, through the analysis of truffle mycorrhizae, the botanist Albert Bernhard Frank revealed in 1885 the mutualistic nature of the interaction between ectomycorrhizal fungi and plants^3^.

True truffles are hypogeous fruiting bodies produced by certain lineages within the Ascomycota, in which spores are enclosed within fleshy ascomata. This reproductive strategy is shared by more than 180 species in the family of Tuberaceae^4^, including the prized black (*Tuber melanosporum* Vittad.) and iconic white (*Tuber magnatum* Picco) truffles. For their carbon uptake, these species obligately interact with fine roots of temperate and Mediterranean ectomycorrhizal (EM) trees and shrubs through chimeric structures called ectomycorrhizae. In addition, recent findings emphasized the ability of some EM fungal species, including the black (*T. melanosporum*), the summer (*Tuber aestivum* Vittad.) and the white (*T. magnatum*) truffles, to concomitantly develop endophytic interactions with a wide range of arbuscular mycorrhizal (AM) plants in natural and planted truffle grounds^5,6^. The benefits of this tripartite interaction remain unclear, with initial experiments suggesting mutual advantages for the EM fungus and its host at the expense of the AM plant^7^, while other studies report contradictory results^8,9^. Recent studies also suggest that fungal–plant interactions may extend beyond known EM host trees, opening a window on a global reconsidering of the ecological framework of mycorrhizal fungi^10^. In this respect, *T. melanosporum* (and more broadly the genus *Tuber*) is increasingly considered as a model for EM fungal ecology, as well as for population genetics^11^.

Native to Europe, most cultivated species of true truffles have considerably expanded their geographical range to other continents, including North America, South America, Africa, Australia and New Zealand^12–16^. Nowadays, the most widespread of them, *T. melanosporum*, is cultivated on all continents, and is considered as a major driver of rural development in France, Italy and Spain with high social, economic and environmental impacts on societies^1,17^. France is the historical cradle of truffle cultivation, especially for the black truffle. Thus, the cultivation of *T. melanosporum* probably started in 1808 in Saint-Saturnin-les-Apts (Southern France^18^), when the French farmer Joseph Talon successfully sowed acorns collected in spontaneous truffle grounds to constitute the first productive orchards. Since Talon’s empiricism, truffles gradually entered a phase of proto-domestication, with the development of controlled inoculation during the 20^th^, and the marketing of mycorrhized seedlings of various species in Fagaceae, Betulaceae, Cistaceae and Malvaceae with an economic impact of several hundred million euros when considering the truffle sales^1^.

Although truffles have been the subject of numerous scientific studies, major experimental constraints have long limited experimental approaches. These include difficulties in isolating truffle mycelium under laboratory conditions, the slow development of productive truffle grounds, limited control over ecological parameters in the field, and the impossibility of establishing ex situ experiments in which the fungus can complete its full life cycle due to spatial and temporal requirements linked to host trees. Consequently, empirical knowledge remains a critical component of truffle cultivation. Indeed, the production of truffles remains highly erratic, even in planted orchards^19^, and high variety of cultural practices (pruning, soil tillage, irrigation, etc.) coexist with empirical approaches some of whose mechanisms have recently been demonstrated (e.g., spore dispersal^20^;). Empirical practices are deeply inspired from observation by truffle growers in natural ecosystems, especially in spontaneous truffle grounds where an astonishingly rich body of empirical knowledge has been accumulated since the end of the 18^th^ century. This knowledge has been disseminated through a unique historical corpus of technical and academic documents classified as grey literature, as well as popular and non-academic writings by practitioners, considered as white literature (for a definition of grey and white literature, see^21–23^). Driven by innovations developed in cultivation systems, publications on truffles have been largely shaped by plantation contexts. By contrast, only a small proportion of the corpus, particularly among historical documents, concerns wild truffle grounds, where natural processes govern the dynamics of the ecological system.

One of the most striking components of truffle empirical knowledge is the concept of companion plants that may sustain the development and the fruiting of different truffle species, notably *Tuber melanosporum*^24,25^. This ancient concept stipulates that “*Finally, there are shrubs which, without being producers themselves, so happily support the action of truffle trees, that if they are found in their vicinity, it will always be at the foot of their trunk, or near their roots, that the truffles will come to form*”^26^. Some truffle growers, informed by field observations and historical literature, deliberately conserve and/or introduce so-called companion plants into cultivated orchards. This practice contrasts with intensive orchards, where cultivation focuses on the EM host and removes all plants from the shrub and herbaceous layers, simplifying the plant community.

TuberIndex aims to compile ecological and interaction data from true truffles as a source of knowledge to offer new insights into its ecological niche and to encourage the emergence of innovative cultural practices. Much of this knowledge is preserved in non-indexed grey and white literature, which remains largely underexplored despite its potential to provide valuable insights into truffle biology and ecology, and to support innovative, nature-based approaches to cultivation. The aim of TuberIndex is to preserve and analyse the full spectrum of knowledge, representations and practices surrounding truffles as they appear in the French-speaking world. We deliberately adopted an inclusive approach by compiling all documents focused on truffles or truffle cultivation, regardless of format, disciplinary field, or intended audience.

First, we compiled interaction data within *Tuberaceae* and plants or fungi, for gathering the whole range of their understated interactions that contribute to their ecological niche. The dataset covers a period from the 17^th^ century to the 21^st^ century and includes 493 documents of non-indexed grey and popular literature. From this corpus, we compiled 3,508 records of associations involving 26 truffle taxa, 418 plant taxa and 53 fungal taxa. Each association is compiled with various metadata, including the effect of the interaction on the truffle and the role of the plant interactor. The dataset is stored in Zenodo^27^.

The objectives of the dataset are to (1) report the extraordinary richness of truffle-plant and truffle-fungi interactions accumulated during five centuries in specialized literature, (2) index the corresponding historical and popular literature on true truffles to preserve empiric knowledge, (3) document the role of the so-called companion plants and (4) provide guidelines and references for scientific research, practitioners, and truffle growers.

## Methods

### Data compilation

#### Documentation research

**Fig. 1:**
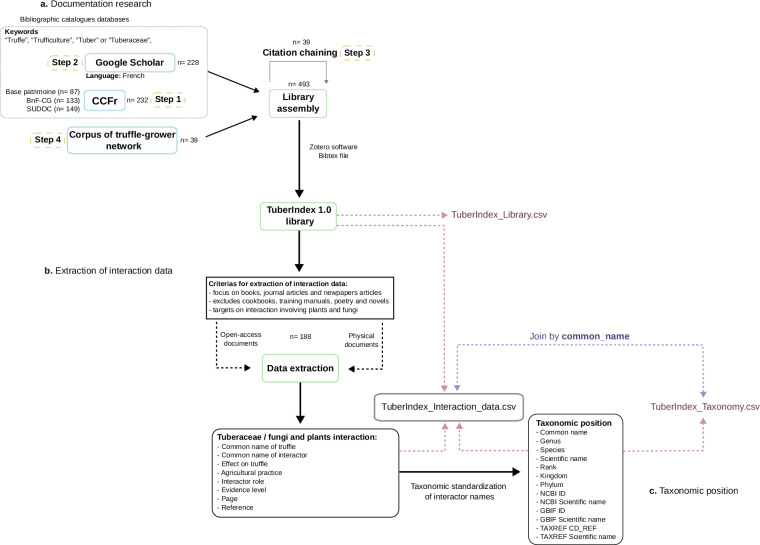
Workflow of TuberIndex 1.0: (**a**) documentation research using a four-step search strategy, (**b**) extraction of interaction data from accessible documents, and (**c**) taxonomic standardization of interactor names.

**Fig. 2:**
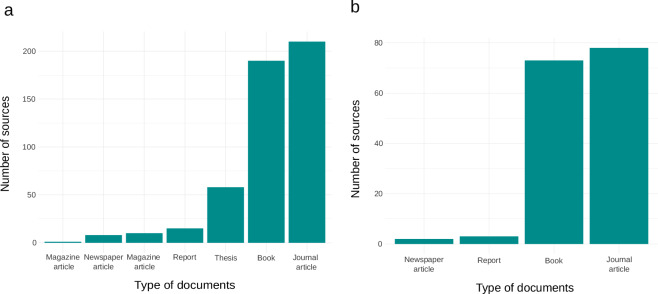
Number of French-language documents identified in TuberIndex 1.0 (**a**) and analyzed for interactions within truffles and plants-fungi in Tuber_index_Interaction_data (**b**).

For assembling *TuberIndex 1.0*, we adopted a four-step searching strategy as developed below (Fig. 1). We focused on books, journal articles, magazine articles, newspaper articles, reports, theses and video records related to truffles and their cultivation, written and/or recorded in French (Fig. 2). Here, we consider true truffles as all species in the genera *Tuber* P. Micheli and *Choiromyces* Vittad., and we exclude from our analysis all other hypogeous Ascomycota, including desert truffles of the genus *Terfezia* Tul. & C.Tul. and all false-truffles (i.e., Glomeromycota and Basidiomycota lineage^28^).

First, we queried the Catalogue collectif de France (CCFr) that compiles the following ten catalog databases: Base Patrimoine, BnF-AM, BnF-CG, CALAMES, CGM, PALME, Rachel, SUDOC, bibliothèques spécialisées de la Ville de Paris and Reseau Valdo. We verified the completeness of our corpus by querying independently BnF-CG, CALAMES, Gallica and SUDOC. On all databases, four independent searches were performed using successively “Truffe”, “Trufficulture”, “Tuber” and “Tuberaceae” as keywords.

Second, we completed our search by including the grey literature published in French and archived in Google Scholar using the same keywords. We specifically aimed at exploiting sources such as periodic newsletters from the sector (both national from the Fédération Française des Trufficulteurs (FFT) and regional) and bulletins from learned societies dealing with mycology and/or botany, and publications in French from journals exploring the anthropological/sociological facets of truffle.

Third, we completed the corpus by conducting a systematic citation chaining on all identified sources in steps one and two. In this third step, the dataset was supplemented by soliciting our network of truffle growers and technicians to help locate additional non-indexed documents.

#### Interaction data extraction

For assembling data interaction of *TuberIndex 1.0*, we collected data in books, journal articles and newspaper articles. Cookbooks, manuals on training truffle dogs, poems and novels listed in Tuberindex 1.0 were excluded prior data extraction (Fig. 2). In upgraded versions of TuberIndex, other types of documents (e.g., magazine articles, newspaper articles, reports, thesis and video recordings) may be mobilized to provide a complementary corpus.

Each accessible online document was analysed for the presence of noted interactions between truffles and plants/or fungi. From *TuberIndex 1.0*, we 1) informatically screened all open-access digitized documents (n = 135) and 2) exhaustively consulted non-digitized material by reading physical materials held in private or public collections (n = 53).

All the names used in the consulted documents to describe the interacting organisms were compiled in a table (*TuberIndex_Interaction_data*). For truffles, the names of species involved in the reported interaction were supplemented by a species name, when possible. If the information provided in the section citing a species, even when analysing the broader context of the section of text concerned, was insufficient to identify the involved species, the identification was limited to the genus *Tuber*. When a given interaction was evoked several times in a document, only the first citation was compiled in *TuberIndex_Interaction_data*. If the author specified an effect of the interaction on truffle development, it was reported as either positive or negative according to the described effect. If the species coexisted with truffles without influencing their development, the effect was reported as neutral.

All associations were recorded along with other contextual metadata, when applicable: page, agricultural practice, interactor role and evidence level. Agricultural practice included all indication of cultural practice and use of all or part of the concerned organism. Concerning the interactor role, we recorded the supposed effect according to the author. In the case of plants, we could distinguish between hosts and companion species. The role of host plant corresponds to a direct and obligate interaction with a physical link established by truffles through mycelia forming either mycorrhizas or parasitic structures or gall formation, this last interaction being considered as unrealistic since^29^. Nevertheless, when gall formation is described in the historic literature, we still categorized it as a host plant interaction because gall formation was previously considered to be a fructification process. In contrast, the role of companion plant corresponds to an indirect and/or facultative, either biotic (e.g., endophytism^30^) or abiotic (e.g., microclimate) interaction between truffles and plants. We added an “evidence level” as a three-scale qualitative indicator distinguishing 1) taxonomically and interaction dubious data due to obvious confusion on the part of the author, from 2) taxonomic and interaction data to be treated with caution due to the presence of unverified and/or unsupported information, and 3) scientifically certified taxonomic and interaction data. In addition, for each record, the bibliographic source and the author who extracted the data were also recorded in *TuberIndex_Interaction_data*.

### Data processing

#### Library metadata compilation

All matched documents were compiled in a bibliographic collection in Zotero (v. 6.0.36), an open-source reference management software for extracted and standardized bibliographic metadata. In Zotero, the source of each record (bibliographic databases, citation chaining, data from the network of truffle-growers), the availability of the document (availability in library collections) were also compiled. This dataset includes all sources, also those that were later discarded (cookbook, novels, poems, manuals on training truffle dogs) for further data extraction.

A CSV file which includes metadata, named *TuberIndex_Library*, was generated by Zotero. To complete this table, two additional metadata columns were added to link the type (*topic*) and the origin (*source)* of the data.

#### Identification of interacting species

The correspondence between the names of the interacting organisms as cited in the literature and their validated taxonomic position (in the binomial nomenclature) was compiled in a new table (*TuberIndex_Taxonomy*). In specific cases, some common names referred to a genus but could be specified at the species rank of the interacting organism using contextual information. For example, some authors used beech (“hêtre” in French) to refer to genus *Fagus*, but in France, only one native species exists, *Fagus sylvatica* L. is natively present. In this case, we used *Fagus sylvatica* for this usual name in a specific column in the table *TuberIndex_Taxonomy* denominated *Probable_species*. This information was completed with an indicator of confidence (*Species_certainty*) and the justification for the chosen correspondence (*Species_comment*). The indicator of confidence, similar as evidence level, is a three-scale qualitative indicator distinguishing 1) ambiguous taxon, hybrid, or lacking sufficient information from 2) common name ambiguous and may refer to multiple species, although one species is predominant in France, and 3) a common name that clearly refers to a single, widely known and dominant native species in France.

All scientific names were matched with NCBI Taxonomy^31^, GBIF Backbone Taxonomy^32^ and TAXREF^33^ to extract taxonomic identifiers and resolve synonymy issues.

#### Dataset assembling

TuberIndex 1.0 was first compiled into one large dataset organized into three tables (*TuberIndex_Interaction_data*, *TuberIndex_Library*, and *TuberIndex_Taxonomy*). A unique common name for the involved organism was used as a linking key between *Tuber_Interaction_data* and *TuberIndex_Taxonomy* (Fig. 1).

### Possible applications

From a fundamental perspective, the assembled corpus constitutes a fully usable resource that can be mobilized by ethnobotanists, community ecologists and functional ecologists. For instance, recorded interactions between truffle species and plants offer great potential to explore the biotic facet of the fundamental niche of emblematic cultivated truffles^34^, and to improve our understanding of the underexplored links between EM and AM plants through below-ground interactions in Mediterranean ecosystems^35^.

From an operational perspective, TuberIndex is a dataset with multiple applications for actors in the truffle sector, including truffle growers, specialized nurseries, companies and farmers. Indeed, it provides a contextualized view (from historical, geographical and ecological aspects) of the diversity of organisms that co-occur with true truffles and their supposed effect on the establishment and/or reproduction of the fungal species. Consequently, the database constitutes a readily available source of information for testing innovative farming practices.

### Perspectives for dataset improvement

We invite scientists as well a larger public range of actors to further enrich the dataset with original documents and extracted data by contacting the corresponding author, in line with the future perspectives of the dataset.

#### Open access

The library of TuberIndex will be hosted by La source portal of Institut Agro of Montpellier (https://institut-agro.docressources.fr/), to provide an open access to documents and information on their physical availability. When possible, all physical public domain documents have been scanned and uploaded to the La Source portal to ensure long-term accessibility.

## Data Records

TuberIndex 1.0 dataset is available at Zenodo^27^ and consists of three UTF-8 encoded CSV files, complemented by a BibTeX export and a raw archive to ensure transparency and reproducibility. The CSV files are organized as follows: (1) *TuberIndex_Interaction_data.csv*, interaction data between Tuberaceae and plants/fungi extracted from the documents, (2) *TuberIndex_Library.csv*, a compilation of bibliographic metadata for all identified documents, (3) *TuberIndex_Taxonomy.csv*, a taxonomic reference for the common names used in the documents.

**Fig. 3:**
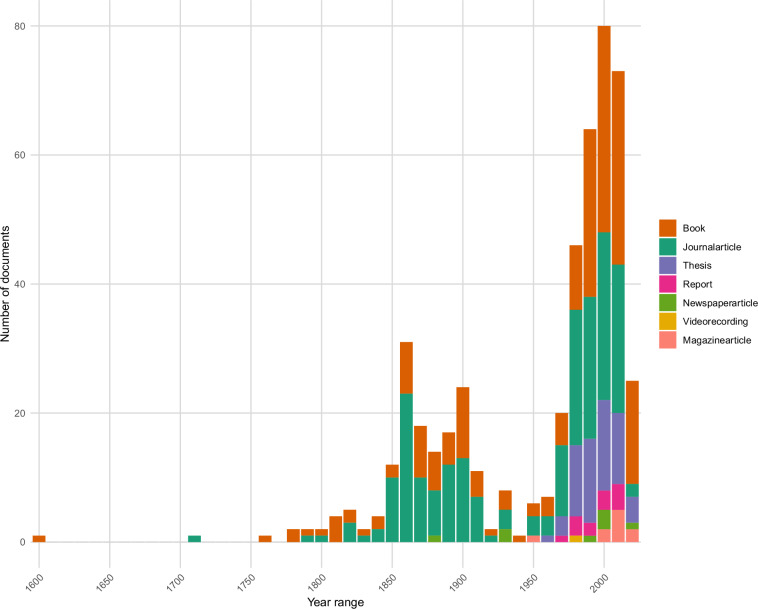
Temporal distribution of French-language documents identified in TuberIndex 1.0 (in ten-year intervals). Colors correspond to different types of documents.

**Fig. 4:**
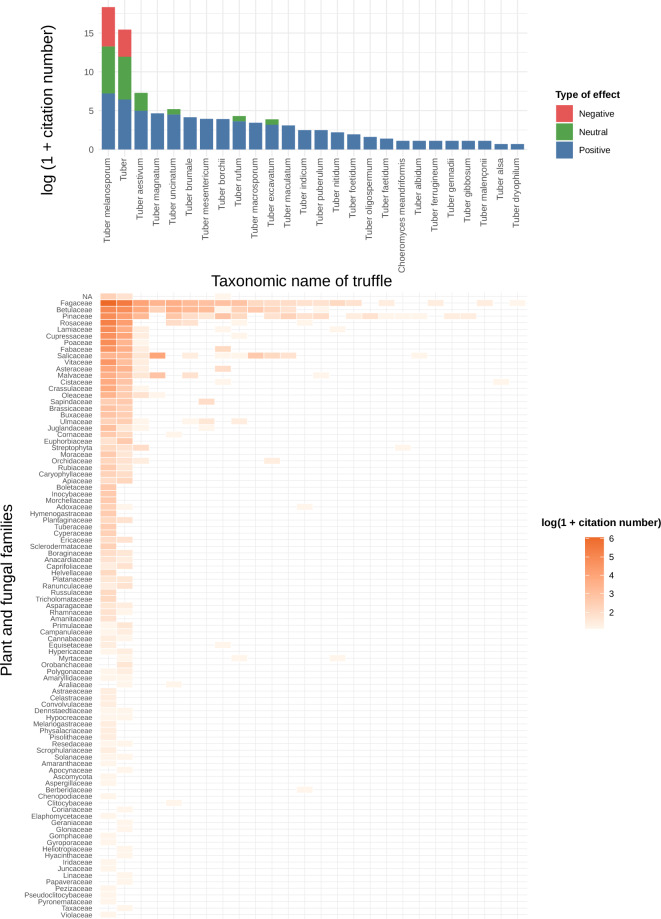
Associations between truffles and plant or fungal families. Bar plots (top) show the number of reported associations per truffle taxon and the type of effect (positive, neutral, negative). The heatmap (bottom) details the distribution of associations across truffle taxa and plant/fungal families, with color intensity indicating the log-transformed number of citations.

The dataset TuberIndex 1.0 bibliographic collection includes 493 documents in the French language (Fig. 2), focused on truffles, ranging from 17^th^ century to 21^st^ century (Fig. 3). For interaction data, 188 documents were examined, resulting in 3,508 observations concerning 26 truffle taxa from one hand (including the genus *Tuber*, 24 species and 1 subspecies), and 418 plant taxa (including 379 Angiosperms, 29 Gymnosperms, 2 Pteridophytes and 8 Plantae) and 53 fungal taxa (including 19 Ascomycota and 34 Basidiomycota) on the other hand (Fig. 4). The taxonomic data includes 1,283 names cited in the literature, each matched with a corresponding scientific name.

File 1: TuberIndex_Interaction_data.

- truffle_common_name: common name used for the truffle.
- truffle_species: scientific species name of the truffle.
- interactor_common_name: common name used for the interacting organism (plant or fungus).
- standard_scientific_name: scientific name standardized according to NCBI Taxonomy and consistent with the TuberIndex_Taxonomy table.
- standard_scientific_name_corrected: corrected scientific name corresponding to the probable species, standardized according to NCBI Taxonomy and consistent with the TuberIndex_Taxonomy table.
- standard_species_certainty: level of confidence in the probable species attribution, as defined in the TuberIndex_Taxonomy table. Categories: high, medium, low.
- effect_on_truffle: reported effect of the interaction on truffle development. Categories: *Negative*, *Neutral* or *Positive*.
- agricultural_practice: agricultural practice associated with the interaction.
- interactor_role: role of the interacting organism according to author(s).
- evidence_level: index indicating the level of confidence attributed to the author(s).
- reference: bibliographic reference formatted according to Nature style, corresponding to the source document from which the interaction is reported.
- page: number of pages in the document where the interaction is reported.

File 2: TuberIndex_Library.

- item_type: type of document.
- publication_year: year of publication.
- author: author(s) of the document.
- of the document.
- publication_title: for publication in journals, name of the journal.
- pages: for publication in journals, name of the journal, page range in the journal.
- issue: issue number of the journal.
- volume: volume number of the journal.
- series: book series name, if applicable.
- publisher: for book or thesis, the name of the publisher.
- language: language of the document (ISO 639-1 code).
- thesis_type: for thesis, the type of thesis. Categories: *Professional doctoral thesis*, *PhD thesis* or *Master’s thesis*.
- available: location or source where the document is available.
- source: origin of the documentation search.
- topic: main topic of the document. Categories: *cookbook*, *novel*, *poem*, *taxonomy* or *ecology, training manual and truffle growing*.

File 3: TuberIndex_Taxonomy.

- common_name: common name used for the interacting organism.
- genus: genus inferred from the common name.
- species: species inferred from the common name.
- scientific_name: scientific name inferred from the common name.
- probable_species: most probable species corresponding to the common name.
- species_certainty: indication of certainty in the probable species attribution. Categories: high, low and medium.
- species_comment: explanation for the species approximation.
- rank: taxonomy rank.
- kingdom: kingdom classification.
- phylum: phylum classification according to the NCBI taxonomy.
- family: family classification according to the NCBI taxonomy.
- ncbi_tax_id: taxon ID according to the NCBI taxonomy.
- ncbi_scientific_name: scientific name from the NCBI taxonomy.
- gbif_tax_id: taxon ID according to the GBIF Backbone Taxonomy.
- gbif_scientific_name: scientific name from the GBIF Backbone Taxonomy.
- taxref_tax_id: taxon ID according to TAXREF (equivalent to CD_REF).
- taxref_scientific_name: scientific name from TAXREF (equivalent to CD_NOM).
- ncbi_tax_id_corrected: corrected taxon ID for the probable species according to the NCBI taxonomy.
- ncbi_scientific_name_corrected: corrected scientific name for the probable species according to the NCBI taxonomy.
- gbif_tax_id_corrected: corrected taxon ID for the probable species according to the GBIF Backbone Taxonomy.
- gbif _scientific_name_corrected: corrected scientific name for the probable species according to the GBIF Backbone Taxonomy.
- taxref_tax_id_corrected: corrected taxon ID for the probable species according to TAXREF (equivalent to CD_REF).
- taxref _scientific_name_corrected: corrected scientific name for the probable species according to TAXREF (equivalent to CD_REF).

## Technical Validation

Data quality from the literature is addressed through the *evidence_level* variable in the *TuberIndex_Interaction_data* table. In addition to the *TuberIndex_Library* table, bibliographic metadata is also available in BibTeX format to facilitate citation and reference management. In the *TuberIndex_Taxonomy* table, truffle names with uncertain taxonomical assignment, potentially referring to multiple species, were assigned to the genus *Tuber*. When the term “truffe” was used in a document without species-level context, it was likewise classified under the genus *Tuber*. The authors validated the correspondence between vernacular and scientific names. Ambiguous cases were reviewed by experts. The list of identified taxa was cleaned and harmonized using three major taxonomic repositories: NCBI Taxonomy, GBIF Backbone Taxonomy, and TAXREF. Synonymies were solved by matching each taxon with its standardized identifier and scientific name across these databases. The correspondence between vernacular names, inferred scientific names, and their corresponding taxon IDs was systematically verified.

## Data Availability

The TuberIndex 1.0 dataset is openly available on Zenodo^27^. It includes three main CSV files (bibliographic data, interaction data, and taxonomy reference), complemented by a BibTeX export and a raw archive. The dataset is released under a CC BY 4.0 licence.

## Code availability

Data processing was realized with R version 4.4.2^36^, and *tidyverse*^37^, *rgbif*^38^, *taxize*^39^ packages. The computer codes used to generate and clean TuberIndex dataset are freely available at: https://github.com/Montan-Gautier/TuberIndex-1.0.
